# Selective reduction of IFN-γ single positive mycobacteria-specific CD4+ T cells in HIV-1 infected individuals with latent tuberculosis infection

**DOI:** 10.1016/j.tube.2016.07.018

**Published:** 2016-12

**Authors:** Catherine Riou, Rubina Bunjun, Tracey L. Müller, Agano Kiravu, Zekarias Ginbot, Tolu Oni, Rene Goliath, Robert J. Wilkinson, Wendy A. Burgers

**Affiliations:** a Division of Medical Virology, Department of Pathology, University of Cape Town, Cape Town, South Africa; b Institute of Infectious Disease and Molecular Medicine, University of Cape Town, Cape Town, South Africa; c Clinical Infectious Diseases Research Initiative, Institute of Infectious Disease and Molecular Medicine, University of Cape Town, Cape Town, South Africa; d Division of Public Health Medicine, School of Public Health and Family Medicine, University of Cape Town, South Africa; e The Francis Crick Institute Mill Hill Laboratory, London, United Kingdom; f Department of Medicine, Imperial College London, United Kingdom

**Keywords:** HIV, *Mycobacterium tuberculosis*, LTBI, PPD, BCG, ESAT-6/CFP-10, Polyfunction, Memory

## Abstract

HIV-1 is recognized to increase the risk for tuberculosis even before CD4+ T cell deficiency is profound. To better understand how HIV-1 alters immunity to latent tuberculosis, we compared the magnitude and functional profile of mycobacteria-specific CD4+ T cells between HIV-uninfected and HIV-infected individuals, using flow cytometry. In HIV-1 infection, IFN-γ single positive mycobacteria-specific CD4+ T cells were decreased, while the frequency of polyfunctional cells (IFN-γ+IL-2+TNF-α+) remained unchanged. Moreover, the proportion of IFN-γ single positive cells correlated inversely with viral replication. Our results suggest that HIV-1 affects mycobacteria-specific cells differentially, depending on their functional capacity.

## 1 Introduction

It is estimated that a third of the world's population is latently infected with *Mycobacterium tuberculosis* (Mtb). While in HIV-uninfected persons the risk of progression from latent to active tuberculosis (TB) is 2–10% in a lifetime, it increases up to 5–10% annual risk in HIV-infected individuals. In order to understand the mechanisms involved in the maintenance and/or impairment of TB latency, it is therefore of interest to define in detail the extent to which HIV affects TB immune responses. The most obvious immune defect caused by HIV is a progressive reduction in absolute CD4+ T cell numbers that correlates with increasing TB disease risk [1], attesting to the critical role of CD4+ T cells for efficient immune responses. Several studies provide compelling evidence that HIV decreases the frequency of peripheral Mtb-specific CD4+ T cells even during the early phase of HIV infection [2], [3], [4]. It has been proposed that, notwithstanding overall CD4+ T cell depletion, HIV may also induce qualitative changes in CD4+ T cell function, further weakening protective immune responses to Mtb. Alteration of the polyfunctional capacity [5], [6], memory profile [7] and lineage differentiation [8] of Mtb-specific CD4+ T cells have been reported. Most work has reported the effect of HIV on TB immune response during active TB; and fewer studies have compared the attributes of mycobacterial-specific CD4+ T cells in the context of latent tuberculosis infection in HIV-uninfected and HIV-infected persons [2], [3], [8], [9].

To better understand the specific qualitative and quantitative deficits affecting immunity in latent TB during HIV infection, we compared the magnitude, functional and memory profiles of CD4+ T cell responses to distinct mycobacterial antigens (ESAT-6/CFP-10 peptide pool, purified protein derivative (PPD) or Bacille Calmette Guérin (BCG)) in HIV-uninfected individuals and antiretroviral therapy naïve HIV-infected individuals with well-preserved CD4 counts.

## 2 Material and methods

### 2.1 Study subjects

Study participants (n = 49) were recruited from Khayelitsha in Cape Town, South Africa, 25 of whom were HIV-uninfected (median age: 23, 60% female), and 24 were HIV-infected and antiretroviral therapy naïve (median age: 31; 96% female; median CD4 count: 625, IQR [545–786]; median HIV viral load: 7788, IQR [3251–17623]). A summary of the participants' clinical characteristics is presented in Table 1. All participants were Mtb-sensitized, as defined by a positive IFN-γ release assay (Quantiferon™ in-tube, Cellestis). None of the participants reported active TB within the eight years prior to their participation in this study. Of note, whilst gender and age distribution differed between the two groups, no significant differences in the magnitude or profile of mycobacteria-specific CD4+ T cells were observed between males and females, or according to age (data not shown).

### 2.2 Blood collection, whole blood stimulation and staining

Peripheral blood was collected in sodium heparin tubes and processed within 4 h of collection. A whole blood assay was performed according to the protocol optimized by Hanekom et al. [10]. Briefly, whole blood (250 μl) was incubated at 37 °C for a total of 12 h with mycobacterial antigens, namely ESAT-6/CFP-10 peptide pool (4 μg/ml), PPD (20 μg/ml, SSI) or BCG (MOI of 4, *Mycobacterium bovis* Danish strain 1331, SSI), in the presence of anti-CD28 and anti-CD49d antibodies. Lyophilized BCG vaccine was resuspended in 250 μl RPMI (Sigma), from which 15 μl (∼3 × 10^5^ organisms) was added to 250 μl of whole blood; this equates to an average multiplicity of infection (MOI) of 4 organisms per monocyte. Brefeldin A was added 7 h after the onset of the stimulation, as per Hanekom et al. [10], as prolonged incubation prior to Brefeldin A addition improved the detection of cytokine responses to complex mycobacterial antigens. Non-stimulated (NS) cells were incubated with CD28 and CD49d antibodies only. At the end of the incubation, red blood cells were lysed with Alternative Lysing solution (150 mM NH_4_Cl, 10 mM KHCO_3_, 1 mM Na_4_EDTA). Cells was subsequently stained with ViViD (Molecular Probes), fixed with FACS Lysing Buffer (BD) and cryopreserved in 10% DMSO in FCS for later batch staining. After thawing, cells were washed, surface stained with CD4-PE-Cy5.5 (S3.5; Invitrogen), CD8-Qdot705 (3B5; Invitrogen), CD27-PE-Cy5 (1A4CD27; R&D Systems) and CD45RO-ECD (UCHL1; R&D Systems), permeablized and stained intracellularly with CD3-APC-H7 (SK7, BD), IFN-γ-Alexa700 (B27, BD), IL-2-APC (MQ1-17H12, BD), IL-17-Alexa488 (N49-653, BD) and TNF-α-PE-Cy7 (MAb11; eBiosciences).

### 2.3 Data analyses and statistics

Cells were acquired on a BD Fortessa and analyzed using FlowJo (TreeStar) and Pestle and Spice software. A positive cytokine response was defined as at least twice the background and data are reported after background subtraction. The gating strategy is presented in Supplemental Figure 1. For statistical analysis of Spice data, we used the statistic tools integrated in the software, where the applied test has the ability to compare multi-component measurements by reducing the comparison to a single test rather than comparing individual components that would require a correction for multiple comparisons [11]. All other statistical comparisons were performed in GraphPad Prism. Univariate statistics were applied and no adjustments were made for multiple comparisons. Non-parametric tests were used for all comparisons (Mann–Whitney U, Wilcoxon Signed Rank or Kruskal–Wallis ANOVA tests). Correlations were performed using the Spearman Rank test. A *P*-value of <0.05 was considered statistically significant.

### 2.4 Ethical approval

Ethical approval for the study was obtained from the University of Cape Town Research Ethics Committee (158/2010). All participants provided written informed consent.

## 3 Results

**Figure 1:**
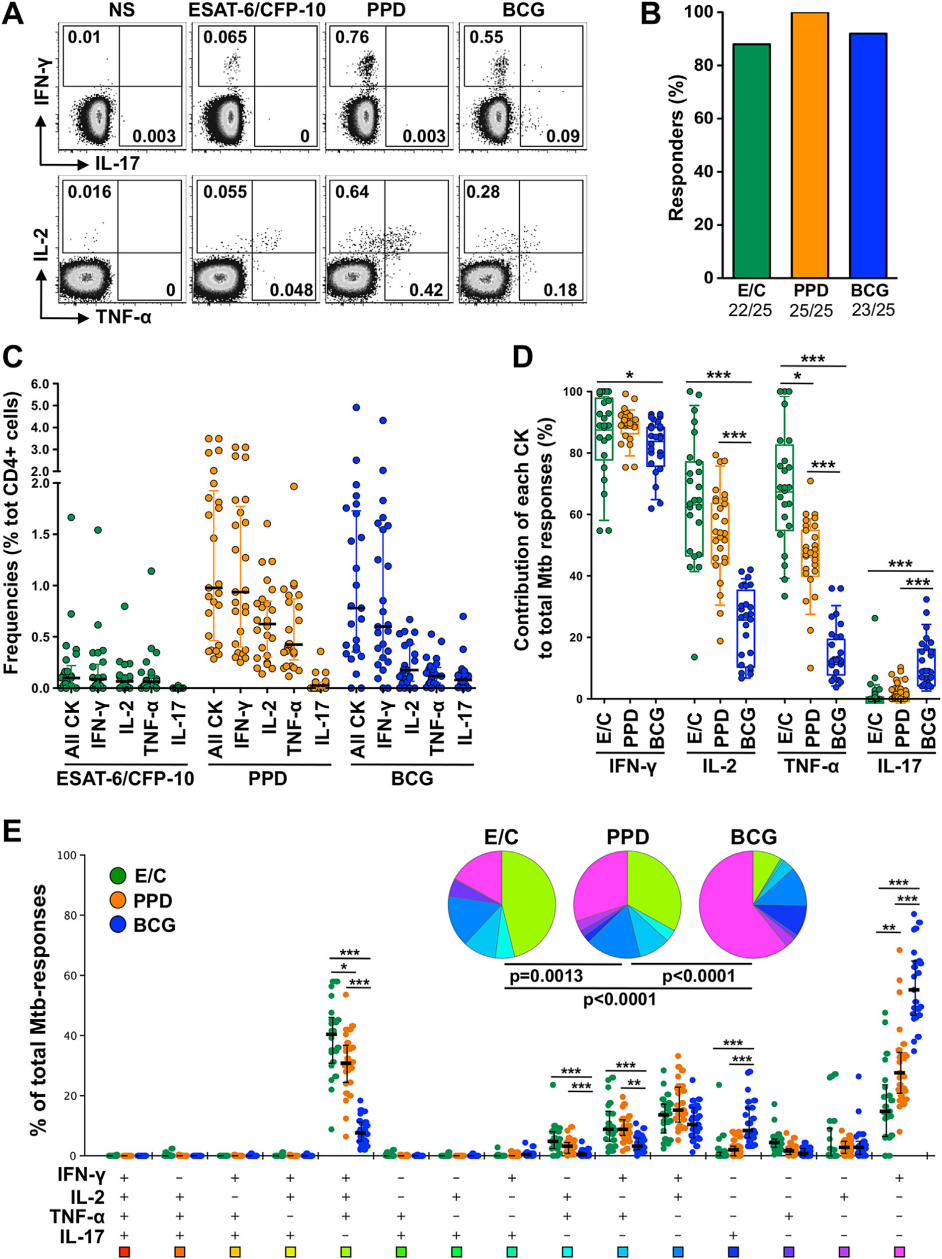
Comparison of the frequency and functional profile of ESAT-6/CFP-10-, PPD- and BCG-responding CD4+ T cells in HIV-uninfected individuals with latent tuberculosis infection (n = 25). *A*, Representative dot plots of IFN-γ, IL-2, TNF-α and IL-17 expression in response to ESAT-6/CFP-10 (E/C) peptide pool, PPD and BCG. NS: Non-stimulated. Numbers represent the frequencies of cytokine-producing cells expressed as a percentage of the total CD4+ T cell population. *B*, Proportion of responding subjects to each antigen. *C*, Frequencies of the overall cytokine responses (All CK) and individual cytokines in response to ESAT-6/CFP-10, PPD and BCG. Bars represent the median and interquartile range (IQR). *D*, Contribution of each cytokine measured to the total mycobacteria-specific response. The median and IQR are shown. Statistical comparisons were performed using a one-way Anova Kruskal–Wallis test. *E*, Proportion of mycobacteria-specific CD4+ T cells producing any possible combinations of IFN-γ, IL-2, TNF-α or IL-17. Horizontal bars represent the median and IQR. Statistical analysis was performed using a Mann–Whitney test. Each slice of the pie corresponds to a distinct combination of cytokines. A key to colors used in the pie charts is shown at the bottom of the graph. ***p < 0.001, **p < 0.01, *p < 0.05.

We compared the frequency, polyfunctional and memory profile of ESAT-6/CFP-10-, PPD- and BCG-responding CD4+ T cells in Mtb-sensitized HIV-uninfected individuals (n = 25) and HIV-infected subjects (n = 24) with well-preserved CD4 counts (median 625 cells/mm^3^). In order to evaluate the impact of HIV infection on mycobacteria-specific responses, it was important to first define and compare the profile of mycobacteria-specific CD4+ T cells in response to distinct antigen formulations commonly used to assess TB immune responses, such direct comparisons not having been reported to date. Thus, we first compared the magnitude and polyfunctional profile of mycobacteria-specific CD4+ T cells in HIV-uninfected individuals in response to an ESAT-6/CFP-10 peptide pool, Mtb PPD or BCG. Figure 1A shows representative plots of cytokine production (IFN-γ, IL-17, IL-2 and TNF-α) in CD4+ T cells in response to ESAT-6/CFP-10, PPD or BCG in one HIV-uninfected participant. All individuals tested had a positive response to PPD; 22 and 23 individuals had a detectable response to ESAT-6/CFP-10 and BCG, respectively (Figure 1B). The overall median frequencies of PPD- and BCG-specific CD4+ T cells were 0.98% (IQR: 0.46–1.9) and 0.78% (IQR: 0.35–1.7) of total CD4+ T cells, respectively (Figure 1C); while ESAT-6/CFP-10 responses were approximately 10-fold lower (median 0.09%, IQR: 0.03–0.22). Analysis of the contribution of each cytokine measured to the overall mycobacterial response (Figure 1D) showed that whilst IFN-γ encompasses more than 80% of the total response to ESAT-6/CFP-10, PPD or BCG, the contribution of IL-2, TNF-α and IL-17 were significantly different amongst the stimuli tested. The response measured to BCG was characterized by a significantly lower contribution of IL-2 (median: 27%) and TNF-α (median: 13%) to the total cytokine response compared to PPD (IL-2: 54%, TNF-α: 48%) and ESAT-6/CFP-10 (IL-2: 65%, TNF-α: 69%). On the contrary, IL-17 production, representing 9% of total BCG responding cells, was rarely detected in response to ESAT-6/CFP-10 or PPD, contributing to less that 1% of the overall response to these antigens. Using a Boolean gating strategy, we further compared the polyfunctional profile of mycobacteria-specific CD4+ T cells. The cytokine production profile of CD4+ T cells varied significantly according to the antigen formulation used. ESAT-6/CFP-10-specific responses consisted predominantly of polyfunctional cells (IFN-γ+IL-2+TNF-α+, median: 40.5%, IQR: 31-46), while BCG responses were mostly producing IFN-γ only (55.2%, IQR: 47-65); PPD-responsive cells exhibited an intermediate profile with comparable proportions of IFN-γ+IL-2+TNF-α+ and IFN-γ single positive cells (31%, IQR: 24–37 and 27.8%, IQR: 21–34; respectively) (Figure 1E). Furthermore, assessment of the memory differentiation profile of mycobacteria-specific responses, based on CD45RO and CD27 expression, showed that mycobacteria-specific CD4+ T cells exhibited mainly an early-differentiated phenotype (∼80%, CD45RO+CD27+). Only subtle differences were observed between stimuli, where ESAT-6/CFP-10-specific CD4+ T cells showed an enriched proportion of naïve-like cells (∼10%, CD45RO-CD27+) compared to PPD and BCG responses (Supplemental Figure 2A and B).

**Figure 2:**
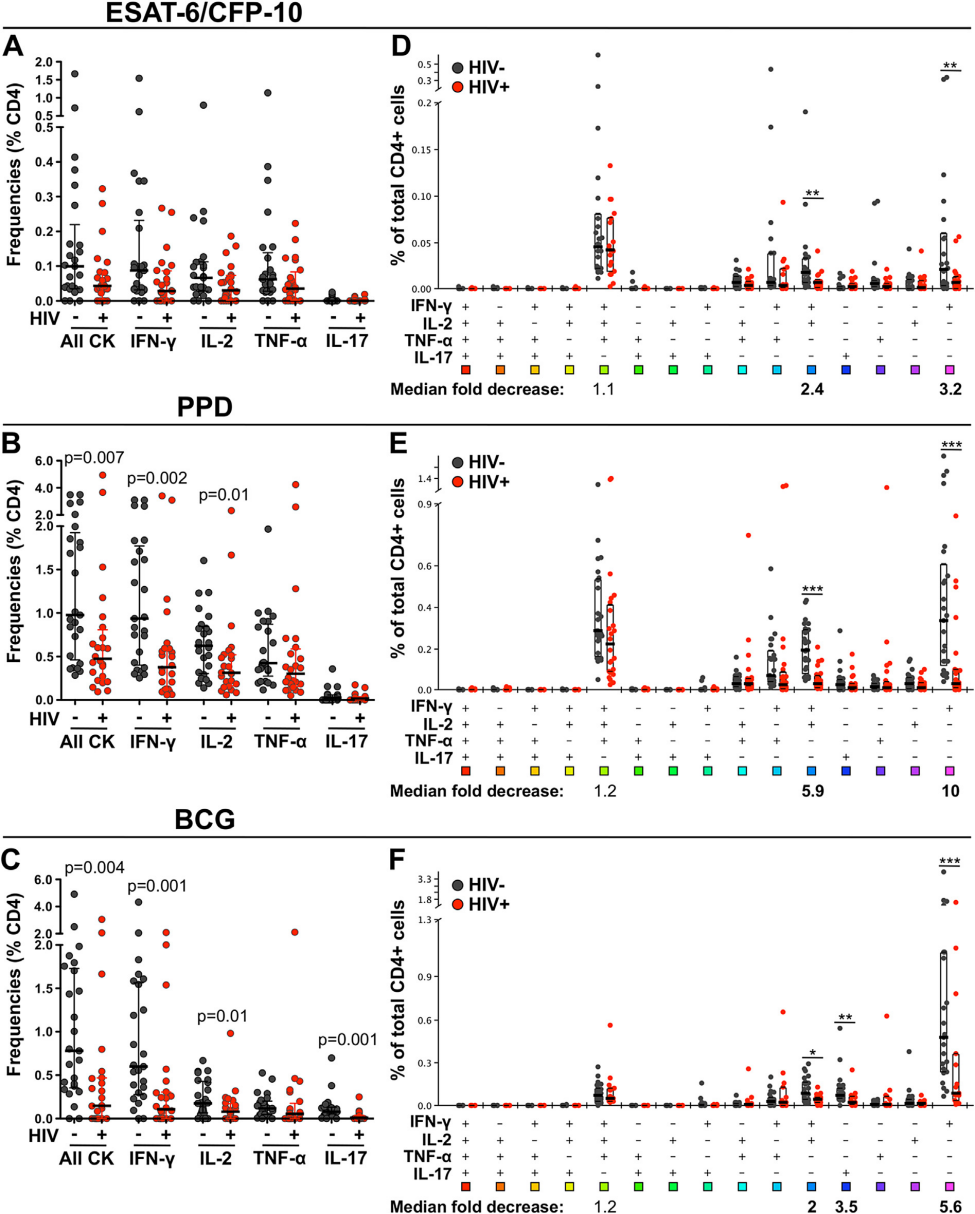
Comparison of the frequencies of ESAT-6/CFP-10-, PPD- and BCG-responding CD4+ T cells between HIV-uninfected (n = 25) and HIV-infected individuals (n = 24). *Left panel*, Frequencies of the overall cytokine response (All CK) and individual cytokines in response to ESAT-6/CFP-10 (A), PPD (B) and BCG (C) in HIV-uninfected (black) and HIV-infected (red) individuals. Bars represent the median and IQR. Statistical comparisons were performed using a non-parametric Mann–Whitney test. *Right panel*, Frequencies of mycobacteria-specific CD4+ T cells producing any possible combinations of IFN-γ, IL-2, TNF-α or IL-17 in response to ESAT-6/CFP-10 (D), PPD (E) and BCG (F) in HIV-uninfected (black) and HIV-infected (red) individuals. Horizontal bars represent the median and IQR. Statistical analysis was performed using the Mann–Whitney test. ***p < 0.001, **p < 0.01, *p < 0.05. (For interpretation of the references to colour in this figure legend, the reader is referred to the web version of this article.)

We next compared the magnitude and functional profile of mycobacteria-specific CD4+ T cells between the HIV-uninfected and HIV-infected groups. The frequency of responders was lower in the HIV-infected group in response to ESAT-6/CFP-10 (18/24) and BCG (15/24; data not shown). It is worth noting that the magnitude of IFNγ+ ESAT-6/CFP-10-specific CD4+ T cells measured by flow cytometry correlated with the IGRA response for both HIV-uninfected (p < 0.0001, r = 0.74) and HIV-infected individuals (p < 0.0001, r = 0.75, data not shown); and there was no difference in the magnitude of IGRA responses between the HIV-uninfected and the HIV-infected group (p = 0.66, data not shown). While no differences were observed for the magnitude of ESAT-6/CFP-10 responses between the groups, the overall magnitude of PPD- and BCG-specific responses was significantly lower (median 2-fold decrease) in HIV-infected compared to uninfected subjects (p = 0.007 and 0.004, respectively) (Figure 2A, B and C).

In depth analysis of the effect of HIV infection on the frequency of mycobacteria-specific cells producing distinct cytokine combinations revealed a predominant reduction of IFN-γ single positive cells, for all antigens, in HIV-infected participants (median fold reduction of 3.2, 10 and 5.6 for ESAT-6/CFP-10, PPD and BCG, respectively) as well as a lower frequency of IFN-γ+IL-2+ cells (median fold reduction of 2.4, 5.9 and 2 for ESAT-6/CFP-10, PPD and BCG, respectively). The proportion of IFN-γ single positive cells in response to each mycobacterial antigen associated negatively with HIV plasma viral load (BCG: p = 0.007, r = −0.66; PPD: p = 0.034, r = −0.43; ESAT/CFP: p = 0.039, r = −0.48; Supplemental Figure 3), with no association observed for the proportion of mycobacteria-specific cells co-expressing IFN-γ and IL-2 or the overall frequency of mycobacterial-responding cells (data not shown). Additionally, the magnitude of BCG-responding IL-17+ cells was also significantly reduced in HIV-infected individuals compared to the uninfected group (p = 0.001). Interestingly, no changes were observed in the magnitude of polyfunctional cells (producing IFN-γ+IL-2+TNF-α+) between the two groups for any of the antigens (Figure 2D, E and F). When evaluating the proportion (rather than frequency) of cytokine combinations contributing to the response, there appeared to be a significantly greater proportion of polyfunctional cells to PPD and BCG in HIV infection (Supplemental Figure 4). These changes in the functional profile of mycobacteria-specific CD4+ T cell in HIV-infected individuals do not however reflect an increase of the polyfunctional capacities of these cells, but are merely a consequence of the specific contraction of IFN-γ single positive CD4+ T cells. Finally, while no differences in the memory profile of CD4+ T cells to the different mycobacterial stimuli were observed between HIV-uninfected and HIV-infected individuals, IFN-γ single positive cells were enriched in the late differentiated subset compared to polyfunctional cells in both groups (Supplemental Figure 2C and D).

## 4 Discussion

The attributes required of Mtb-specific CD4+ T cells to efficiently maintain TB latency remain unclear. Studying populations with elevated risk of TB infection and/or reactivation, such as HIV-infected individuals, may help shed light on the mechanisms involved in the heightened risk of TB disease in HIV-infected individuals with latent infection, even before CD4 depletion is profound.

We compared the magnitude and functional profile of CD4+ T cells in response to ESAT-6/CFP-10 peptides, PPD and BCG in HIV-uninfected and HIV-infected individuals. Our results confirm that in HIV infection, depletion of CD4+ T cells specific for mycobacteria occur even before substantial peripheral CD4 cell loss [2], [3]. Our main novel finding was that in HIV-infected individuals, mycobacteria-specific CD4+ T responses were characterized by a selective and prominent decrease in IFN-γ single positive cells, while the magnitude of polyfunctional cells (defined as IFN-γ+IL-2+TNF-α+) remained unchanged compared to HIV-uninfected individuals. Moreover, the proportion of IFN-γ single positive cells correlated inversely with HIV viral load, suggesting that HIV may preferentially deplete this subset. What mechanisms could explain the preferential contraction of certain functional subsets and/or the maintenance of others? Geldmacher et al. showed that the ability of cells to secrete MIP-1β was linked to their resistance to HIV infection [12]. While Mtb-specific CD4+ T cells were poor MIP-1β producers, making them more susceptible to HIV infection, MIP-1β secretion was mostly observed in polyfunctional Mtb-specific cells (∼15%) [3], suggesting that this could contribute, in part, to the maintenance of polyfunctional responses to Mtb during HIV infection. Alternatively, HIV could reduce mycobacterial CD4+ responses by promoting premature exhaustion of specific subsets. In our study, we showed that IFN-γ single positive mycobacteria-specific CD4+ T cells were enriched in a late differentiated memory subset, a subset more susceptible to HIV infection [13]. Moreover, elevated levels of PD-1 have been reported in IFN-γ single positive Mtb-specific CD4+ T cells (compared to more polyfunctional cells) [14], so monofunctional cells could conceivably be more prone to depletion and/or anergy. Further experiments, assessing the expression of β-chemokines, CCR5, inhibitory receptors and pro-survival molecules could help define whether Mtb-specific CD4+ T cells endowed with different cytokine secretion profiles are differentially permissive to HIV and/or exhibit different susceptibility to anergy or survival.

We also found that HIV-induced depletion of mycobacteria-specific CD4+ T cells was greater for complex antigens that need processing to be presented. Since HIV disrupts host antigen presentation mechanisms by promoting the down-regulation of co-stimulatory (CD80 and CD86) and MHC II molecules [15], it could be speculated that alteration of signaling pathways required for optimal CD4+ T cell stimulation could impede recognition of Mtb-specific responses. Further experiments, assessing the processing and presentation of Mtb in the context of HIV infection, will be necessary to explore this hypothesis. Moreover, it has also been reported that HIV induces a marked impairment of IL-12 secretion from macrophages and dentritic cells [16], [17]. Thus, it could be hypothesized that reduced IL-12 production could bias CD4+ T cell lineage commitment, limiting the generation of Th1 cells. Additional experiments will be needed to verify this postulate.

We described a higher proportion of polyfunctional cells in response to Mtb antigens than previously reported [18], which may be a result of continual antigen exposure in high burden settings which could promote maintenance of these cells. Whether polyfunctional cells play a role in Mtb containment or reflect high bacterial exposure remains unclear [18], [19]. This study was limited to assessing Mtb responses in the blood, which do not necessarily reflect the quality and quantity of memory responses present at the site of infection. Moreover, the profile of mycobacteria-specific responses was defined by analyzing four cytokines, which may only partially recapitulate Mtb-specific CD4+ T cell responses.

In conclusion, we found that HIV-1 infection impairs Mtb-specific responses not only by numerically reducing Mtb-specific CD4+ T cells but also alters the balance of their functional profile, and show for the first time the selective depletion of IFN-γ single positive cells. Our results provide new insights into potential mechanisms that perturb immune control of latent TB infection.

## Financial support

This work was supported by the European and Developing Countries Clinical Trials Partnership (EDCTP) grant TA_08_40200_020, the South African Medical Research Council (MRC) and the Poliomyelitis Research Foundation (13/06). CR is funded by the National Institutes of Health, the Office of the Director (OD) (NIH, R21AI115977). RB is a Carnegie Corporation PhD Fellow and also received funding from the University of Cape Town and the Canada Africa Prevention Trials (CAPT) Network. RJW is supported by the Wellcome Trust (084323 and 104803), the Francis Crick Institute (10218), the European Union (FP7-Health-F3-2012-305578), South African National Research Foundation (NRF-SA), Medical Research Council SA-Strategic Health Innovations Partnership and the National Institutes of Health (U19AI1112761). WAB is a Wellcome Trust Intermediate Fellow in Public Health and Tropical Medicine (089832/Z/09/Z). The funders had no role in study design, data collection and analysis, decision to publish, or preparation of the manuscript.

## Figures and Tables

**Figure:**
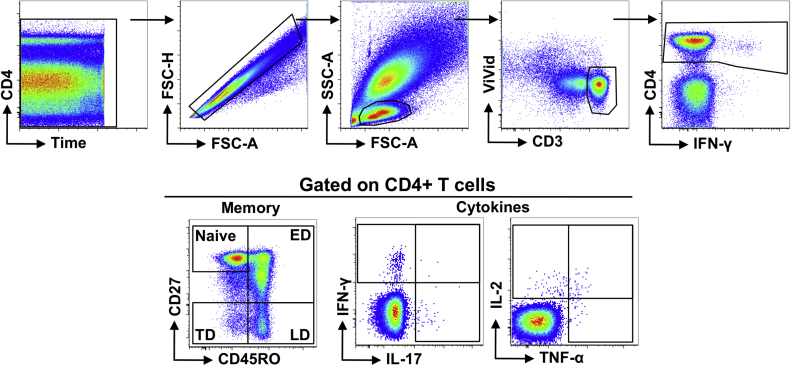


**Figure:**
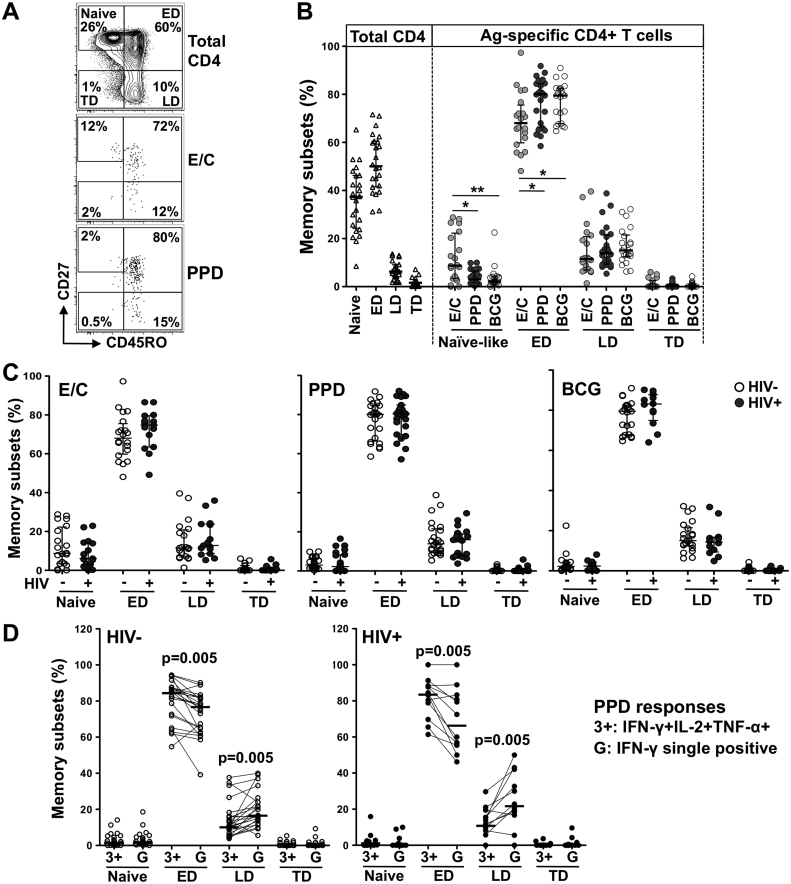


**Figure:**
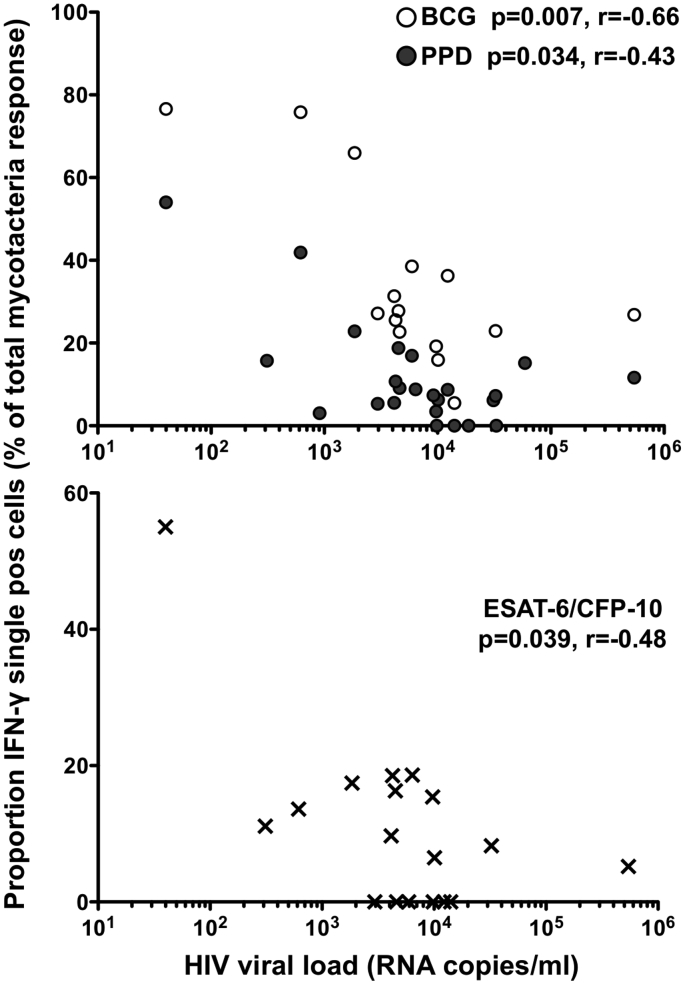


**Figure:**
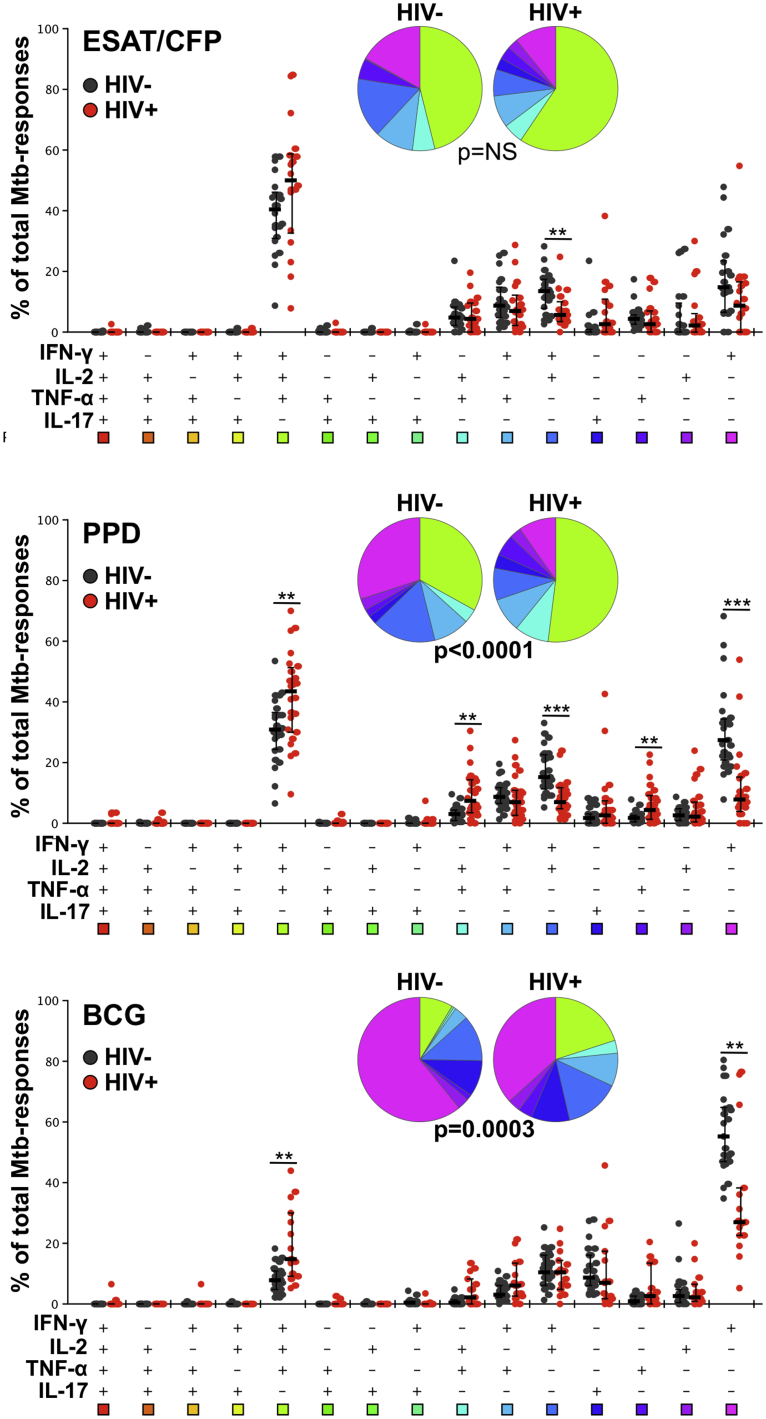


**Table 1:** Clinical characteristics of study participants.

	HIV-uninfected	HIV-infected	*P*-values∗
Number of participants	25	24	
Median CD4 count [IQR]†	813 [675–933]	625 [545–786]	p = 0.003
Median HIV viral load [IQR]§	na	7788 [3251–17,623]	na
Interferon-γ release assay [IQR]∗∗	7.4 [1.1–10.5]	3.0 [1.1–10]	p = 0.73

∗ *P*-values were calculated using a Mann–Whitney t-test.

† CD4 count, measured using a Flow-CARE™ PLG CD4 test (Beckman Coulter), is expressed as cells/mm^3^.

§ HIV viral load, determined using an Abbott m2000 RealTime HIV-1 assay, is expressed as RNA copies/ml.

∗∗ Interferon-γ release assay results are expressed in IU/ml (background subtracted).
